# Can I benefit from laboratory automation? A decision aid for the successful introduction of laboratory automation

**DOI:** 10.1007/s00216-023-05038-2

**Published:** 2023-11-30

**Authors:** Nicole Rupp, Robert Ries, Rebecca Wienbruch, Thole Zuchner

**Affiliations:** 1 Faculty for Life Sciences, Professorship for Bioanalytics and Laboratory Automation, Albstadt-Sigmaringen University, Anton-Günther-Str. 51, 72488 Sigmaringen, Germany; 2 Drug Discovery Sciences, Boehringer Ingelheim Pharma GmbH & Co. KG, Birkendorfer Strasse 65, 88397 Biberach an der Riss, Germany

**Keywords:** Laboratory automation, Liquid handling, Bioanalysis, Laboratory workflow, Assay development, Small- and medium-sized enterprises

## Abstract

The large volumes of samples to be analysed every day would be impossible to manage without laboratory automation. As laboratory procedures have progressed, so have the tasks of laboratory personnel. With this feature article, we would like to provide (bio)chemical practitioners with little or no knowledge of laboratory automation with a guide to help them decide whether to implement laboratory automation and find a suitable system. Especially in small- and medium-sized laboratories, operating a laboratory system means having bioanalytical knowledge, but also being familiar with the technical aspects. However, time, budget and personnel limitations allow little opportunity for personnel to get into the depths of laboratory automation. This includes not only the operation, but also the decision to purchase an automation system. Hasty investments do not only result in slow or non-existent cost recovery, but also occupy valuable laboratory space. We have structured the article as a decision tree, so readers can selectively read chapters that apply to their individual situation. This flexible approach allows each reader to create a personal reading flow tailored to their specific needs. We tried to address a variety of perspectives on the topic, including people who are either supportive or sceptical of laboratory automation, personnel who want or need to automate specific processes, those who are unsure whether to automate and those who are interested in automation but do not know which areas to prioritize. We also help to make a decision whether to reactivate or discard already existing and unused laboratory equipment.

## Introduction

Nowadays, laboratory automation is required in almost every bioanalytical laboratory [1, 2]. In larger laboratories, automation is indispensable due to the overwhelming amount of analytical processes. This includes handling the large number of medical samples in diagnostic laboratories as well as the demand for high-throughput screening in the discovery of new compounds [3–6]. As laboratories have shifted towards automation, the requirements for laboratory personnel have also changed [7, 8].

Large laboratories and companies have dedicated engineering departments which are responsible for automating laboratory processes [1]. These departments offer technical support and oversee the implementation of hardware and software of new equipment into automated process workflow [9]. Financial and personnel capacities are given, to plan and integrate laboratory automation. However, operating the systems remains the responsibility of the scientists and significantly differs from manual laboratory processes [10].

The further we move mentally in the direction of smaller laboratories, the more of these tasks related to laboratory automation falls on the laboratory employees themselves. Smaller and medium-sized laboratories are also under pressure to automate laboratory processes. On one hand, this is driven by the need to remain competitive in the market and, on the other hand, to address the existing shortage of skilled personnel [1, 4, 11]. However, financial resources are often more limited compared to larger companies, and internal technical support dedicated to laboratory automation equipment is less common at this scale [12, 13].

Regardless of the size of the laboratory, laboratory automation is elementary in each of these laboratories. Thus, it requires technical understanding from (bio)chemical practitioners since operating and understanding of the automated process remain for scientists [7]. For instance, programming of laboratory equipment usually addresses the scientists themselves, since they are familiar with the processes in their laboratory. For the example of the liquid handler, this involves the properties of the liquids (liquid class), cross-contamination (necessary cleaning steps) and, last but not least, the experimental procedure itself (protocol steps as well as timing) [2].

A crucial objective for manufacturers of laboratory equipment is to develop software that is as user-friendly as possible. This ensures that scientists feel comfortable programming the execution of their processes without hesitation [14]. Nevertheless, the more complex the laboratory device is, the more complex will its control software be. Therefore, the concept of an amphibious researcher is becoming more and more important [8]. Amphibious researchers — meaning scientists who are familiar with the life sciences (wet) on the one hand and have knowledge of automation (dry) on the other (Mellingwood, 2018) — are required to manage tasks that can only be done with the knowledge of both areas [7, 8]. Mellingwood (2018) describes the need of a new generation of scientists, trained to use laboratory automation systems as naturally as a manual pipette is used today [7]. However, a bioscience study program that also involves engineering content is hard to find [12]. In addition, the cooperation between bioscience and engineering faculties is often not very close [12]. However, operating the laboratory equipment is only one side of the coin.

The gap between scientists — especially in small- and medium-sized or research laboratories — and laboratory automation considers not only their application, but also their necessity and impact on workflows. The market for automation in laboratory is huge, and a decision in favour of an automation system is difficult in many ways. There are many highly specialized laboratory devices that can automate specific processes in laboratories [15]. Usually, the capacity of scientists is too limited to survey this market. They are busy with their demanding activities and do not have time to deal with or feel alone with the topic of laboratory automation [16].

Even when scientists reach out to equipment manufacturers, the representatives often recommend devices highlighting their advantages, driven by their goal to make sales. Therefore, it becomes crucial to carefully consider essential factors and independently evaluate whether a specific system is suitable for the laboratory process and environment in a systematic manner [17].

With this feature article, we want to provide a guide for structured discussion regarding laboratory automation in your laboratory. We write this article from the perspective of a scientist in the field of bioanalytics. We address scientists or other professionals in the field of bioanalytics/bioscience who have little or no experience with automation, in order to give an overview on the topic of laboratory automation. This includes scientists working in what they consider to be a non-automated laboratory and scientists working in a laboratory with (partially) automated processes.

We neither want to convince you to automate processes nor to discourage you to automate processes. Rather, we want to support you in finding the best decision for your laboratory in terms of automating processes. This could also mean that — with the help of this feature article — you might conclude that automation of a certain process in your laboratory does not make sense. Therefore, we will outline several perspectives to consider when discussing laboratory automation. Summarizing, we hope to encourage scientists to become more familiar within the field of laboratory automation.

### How to read this feature article

**Fig. 1:**
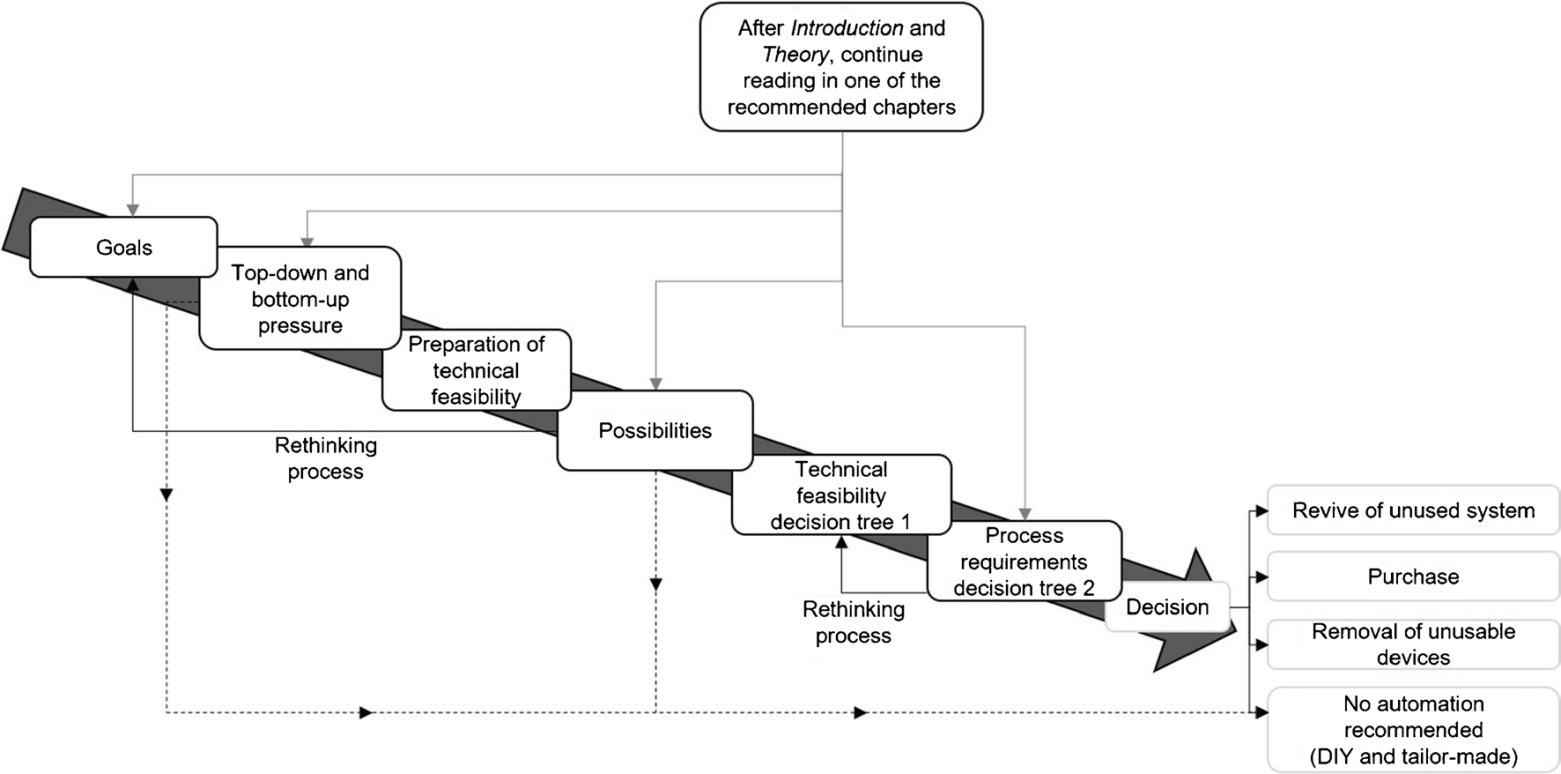
How to read this feature article. After you have read Introduction to Theory, you will get recommendations with which chapter to continue. At certain points, you may be invited to rethink your last decision (“rethinking process”). Alternatively, at a relatively early point in time, you may conclude that no automation is recommended in your specific scenario (dotted arrows)

We structured our article as a guide for the automation of laboratory processes (see Fig. 1) inspired by the gamebooks that were common in the 1980s. Due to the specific needs and desires of different reader groups, we actively encourage the reader not to read every paragraph of this feature article one after the other. Instead, at the end of each chapter, we will guide you to an appropriate next chapter (Fig. 1, light grey arrows) suitable each initial perspectives, facts or goals. Thus, each reader can create an own tailor-made reading flow. We would advise you to make a note of the chapters you read so that you can return to them. Readers who might not have an application but are interested in the topic of laboratory automation are invited to follow the article as “silent readers”.

While reading this article, it is possible that your opinion about laboratory automation will change. On the one hand, you may decide against automation based on our recommendation (Fig. 1, dotted arrows), or you may consider automation as an option and have to re-read some chapters (Fig. 1, rethink process). Consider Fig. 1 as an overview that will serve as a summary at the end of your reading. With our guide, we want to help you to make a decision whether you should automate laboratory processes or not. Please continue reading Theory*.*

## Theory

The benefits of automated processes in life science laboratories have been widely described [1, 7, 10, 12]. Especially in the field of bioanalytics, laboratory automation offers pertinent advantages. Beside the positive economic impact, laboratory automation can reduce random errors and improve time management and bioanalytical parameters. This leads to faster availability of more reliable results.

Holland and Davies (2020) published a review article on this topic. The authors describe the situation of automated processes in research laboratories in great detail. They discuss the advantages, but also the limitations of laboratory automation. If you would like to take a closer look at the topic, we recommend this review [12]. Certainly, we will revisit some aspects in this article.

Presumably, most readers of this feature article will have a positive attitude towards laboratory automation. Some will be enthusiastic about setting up a system in their laboratory. Others are not sure if an automation system is suitable for their laboratory — and they may be right in their expectations. The most important step before investing time and money into laboratory automation is to consider why the automation system is essential or at least meaningful for the specific laboratory situation [10, 18]. There are certainly some good reasons for establishing automation, but there are also a various number of reasons that form the wrong foundation for an automation project [19].

At the end of the year, it often happens that institutions still have funds that need to be spent. Others want to modernize their laboratory and purchase a new product to increase work motivation. These are reasons that usually lead to the purchase of automation systems that end up being unused in the laboratory, taking up valuable lab space, which must be avoided. The conclusion that an automation system is suitable for the own laboratory because it works for other laboratories is also a misconception. Not only the activities of a laboratory are decisive for or against an automation system, many other aspects, such as staff resources, play a role here [20]. In case you are deciding on an automation system during the article, you will find the appropriate counter-questions in later chapters, which you should consult with the relevant departments or persons before any purchase is considered [18]. We also have included helpful procedures in case you already have unused equipment in the laboratory, as described above.

If you think you have — at the moment — well-justified reasons for implementing an automation system, please continue reading Goals. Readers being not sure if their reasons are valid, please read Useful automated processes. We also address readers thinking they might act for wrong reasons (as described in this chapter). In this case, please read Possibilities. Those who already find unused laboratory systems in their lab environment continue reading Preparation for evaluation*.* The silent readers start reading Top-down and bottom-up pressure.

## Goals

Since you think that your reasons are more valid than the negative examples in Theory, you should analyse the precise reasons for your desire to automate [18]. In general, there are two initiatives or categories of reasons for implementing laboratory automation.

If you have no specific process in mind that you want to automate, please follow Useful automated processes*.* In case the automation is a customer or authority requirement, continue reading in Process preparation*.* If the automation of a specific process is your personal request or the request of your colleagues or employees, follow Top-down and bottom-up pressure.

### Useful automated processes

This chapter is for readers who want to automate but have no specific process in mind and for the group of readers who are unsure whether they have valid reasons for laboratory automation (including the “rethinker”). In general, it is not a bad sign being interested in laboratory automation but having no idea whether to automate or what to automate. In this case, you should carefully consider the reasons for your automation desire. There are many examples from the past where purchased automation systems failed, because they could not achieve the desired goals [19, 21]. The reasons for this scenario are various. For example, the automated process does not fit the laboratory’s workflow: If the automation system leads to a higher throughput in one department, the pre- and post-processing (e.g. material preparation and data analysis) in other departments must also match the increased throughput [10]. Another example is that the automated process does not even lead to the desired goal. If you want to improve the throughput, your system should actually result in an increase in throughput rather than, for example, just an improvement in quality.

These examples underline how important a detailed analysis of laboratory processes, workflow and goals is. Keep in mind that investing in an inappropriate system can have far-reaching consequences. Failure could mean that laboratory automation is never considered again for your organization. Therefore, it is important to ensure that the automation goals can be achieved, meaning the goals and reasons for automation should first be identified in the first step. Based on this, it is easier to infer the process to automate [18]. Thus, these are the first steps towards the decision for successful laboratory automation. Please read on in Top-down and bottom-up pressure.

## Top-down and bottom-up pressure

In general, two motives can be observed when laboratory automation is considered [10]. Either the reason (top-down) or the benefit (bottom-up) provides the motivation for the desire to automate a certain process.

You usually act according to the top-down pressure when you want to automate a certain process. The reasons for automation are often related to the characteristics of the process or its impact on the workflow in the laboratory. In other words, your motivation is to improve the current state. Top-down reasons include monotonous, hazardous (infectious samples, toxic or radioactive substances), error-prone or sensitive (e.g. sterile) processes. In addition, impacts on the lab routine are reasons driven by top-down pressure. This refers to complicated processes (many steps, equipment or substances), time-consuming processes and large throughput (many samples less frequently, a few samples more frequently or many samples more frequently).

**Fig. 2:**
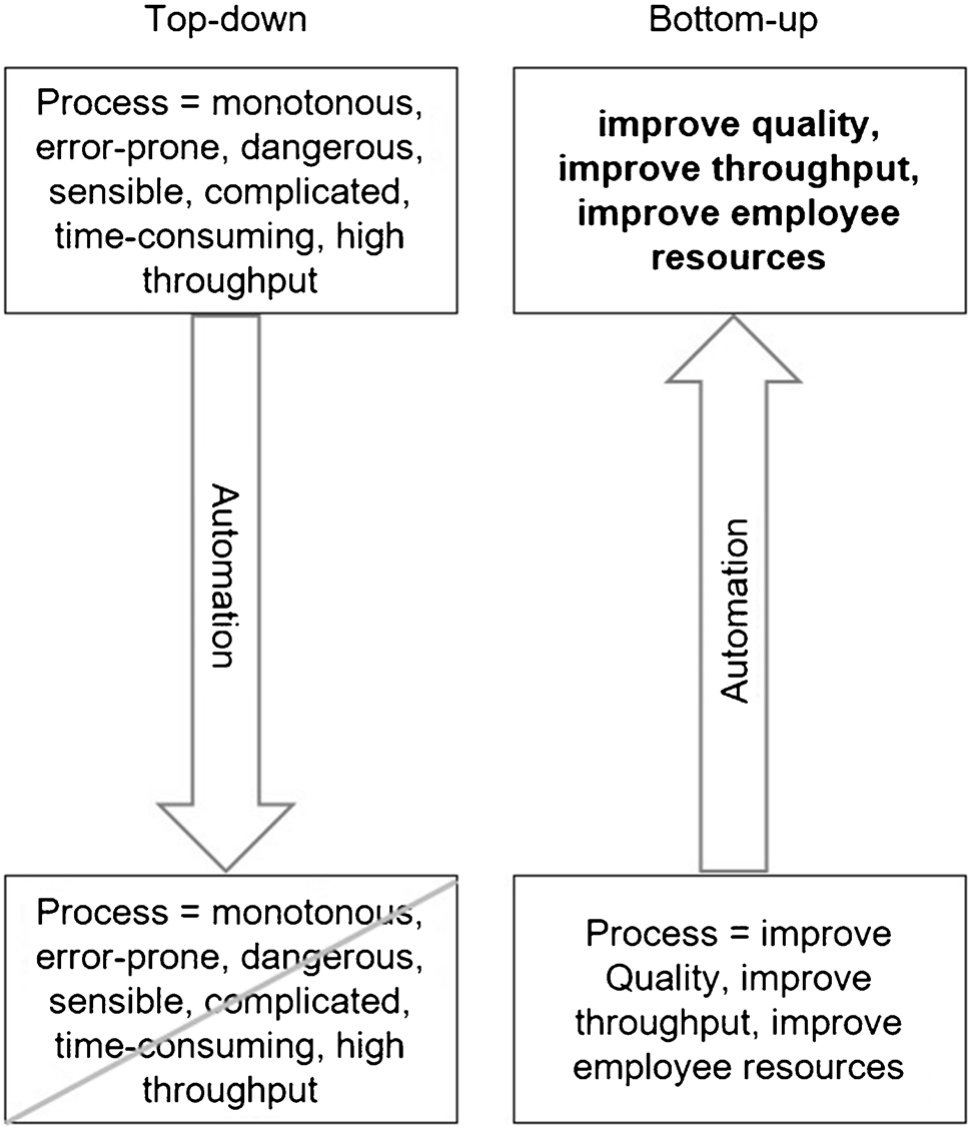
Motivation reasons to automate lab processes. Reasons for the urge of laboratory automation can be initiated by a specific process (top-down) or lead to the process to be automated by a desired goal (bottom-up)

In contrast to top-down pressure, bottom-up is guiding you if you do not want to automate a specific process but rather want to benefit from one or more of the classic advantages of laboratory automation. This means your trigger is to achieve the target state. Reasons for laboratory automation from the bottom-up pressure point of view are quality requirements, improved throughput or time per sample and the resulting availability of personnel [22]. Both automation pressures are visualized in Fig. 2.

Using the example of pipetting assays in microtiter plates, both pressures can be demonstrated. When thinking about laboratory automation, the pipetting process comes to your mind, because it is very time-consuming and error-prone. Therefore, you want the automation to eliminate these properties of the pipetting process (top-down pressure). If you want to improve the quality of the results or achieve a higher throughput and conclude to automate the pipetting process, you act according to the bottom-up pressure. Both initiatives are valid reasons for laboratory automation. The important aspect is to be aware in advance of which goals are being achieved with the automation. This chapter will help you to identify processes that are well suited for automation due to well-justified reasons. If you want to automate a specific process (top-down pressure), please continue with Analyse reasons for a specific process to be automated. Those readers who are driven by the bottom-up pressure and do not know yet which process to automate, continue reading on Identify processes*.* As a silent reader, we recommend reading Analyse reasons for a specific process to be automated.

### Analyse reasons for a specific process to be automated 

Even if it seems trivial to readers who already know which process they want to automate, we would like to recommend them to go through this chapter. As mentioned before, automating processes without solid foundation often leads to failure [19]. Therefore, it is advisable to take a closer look at the process and the reasons for automation [10, 20]. To evaluate your processes and corresponding automation reasons, we developed checklist 1 (Table 1). Insert your processes and rate them depending on the reason (called properties) for their automation. Feel free to add more properties or to delete some of the suggested ones, in case you feel that this reflects your situation in the laboratory in a better way. Please continue reading Additional information for the checklist for additional information before applying checklist 1 (Table 1). Silent reader also follow Additional information for the checklist.

**Table 1:** Checklist 1: Identify the processes and reasons for automation according to top-down scheme. Example is in italic, each process is ranked with a score from 1 (weakest expression) to 10 (strongest expression), and the sum of all assessment categories per process expresses how strong the urge to automate is. In the last column “ranking”, the processes can be numbered in descending order of the sum

Top-down pressure										
Process		Properties							Score	Ranking
No.	Name	Monotonous	Error-prone	Dangerous	Sensible	Complicated	Time-consuming	Throughput		
*01*	*ELISA*	*8*	*10*	*2*	*10*	*5*	*8*	*4*	*47*	*1*
*02*	*Weighing*	*8*	*6*	*4*	*6*	*2*	*3*	*1*	*30*	*4*
*03*	*pH Measurement*	*6*	*2*	*0*	*2*	*2*	*3*	*1*	*16*	*5*
*04*	*Cell passaging*	*7*	*8*	*3*	*8*	*3*	*9*	*7*	*45*	*2*
*05*	*HPLC*	*3*	*6*	*6*	*8*	*6*	*7*	*6*	*42*	*3*
*06*	*…*	*…*	*…*	*…*	*…*	*…*	*…*	*…*	*…*	*…*

### Identify processes

Due to the fact that you focus less on the processes and more on the goals of automation, it is necessary to identify the processes that will lead to the achievement of your automation goals [19]. We developed checklist 2 (Table 2) to help you rate your laboratory processes. Enter your laboratory’s processes in the list and rate each process according to the targeted benefits (called properties) you would achieve through automation. Feel free to add additional properties if they apply to your laboratory. Please continue reading Additional information for the checklist.

**Table 2:** Checklist 2: Identify the processes and reasons for automation according to bottom-up scheme. Example is in italic, each process is ranked with a score from 1 (weakest expression) to 10 (strongest expression), and the sum of all assessment categories per process expresses how strong the urge to automate is. In the last column “ranking”, the processes can be numbered in descending order of the sum

Bottom-up pressure									
Process		Properties							Ranking
No.	Name	Improve quality	Improve throughput	Improve personnel resources	…	…	…	Score	
*01*	*ELISA*	*8*	*7*	*8*	…	…	…	*23*	*2*
*02*	*Weighing*	*1*	*5*	*8*	…	…	…	*16*	*4*
*03*	*pH Measurement*	*1*	*3*	*8*	…	…	…	*12*	*5*
*04*	*Cell passaging*	*9*	*7*	*9*	…	…	…	*25*	*1*
*05*	*HPLC*	*8*	*1*	*8*	…	…	…	*17*	*3*
*06*	*…*	…	…	…	…	…	…	…	…

### Additional information for the checklist

Independently of which checklist (checklist 1 or 2) you use, we want to give you an idea of how your rating could look like. As demonstrated in the examples in checklist 1 and checklist 2, we express the strength of the properties with a scoring system. For example, you can assign a score between 1 and 10 (weakest to strongest) to each process, depending on the strength of each property. The score for each property can be summed up for each process. The highest sum is the process that most urgently needs to be automated. In the last column of each checklist, the processes can be ranked descending, according to their sum (or urge of automation). The ranking enables a direct identification of the laboratory processes that should be automated successively. We show an example distribution of points in both checklists (checklist 1 and 2).

It is also possible to expand the checklists in terms of weighting the properties. For example, properties of the checklists could first be assigned a factor according to their relevance. For checklist 1, that might mean that the categories are assigned the following weights: “monotonous” 0.025, “throughput” 0.05, “time-consuming” 0.1, “complicated” 0.15, “sensitive” 0.2, “error-prone” 0.225 and “dangerous” 0.25, with the sum of all weights resulting in 1.0. This means that if the score is the same in different property categories, the impact will be different due to the weighting. To obtain the final score for each process, two sub-calculations are performed. In the first step, the score of each property is first multiplied by the corresponding weight to calculate the cleaned score. Then, the cleaned scores per process are accumulated resulting in each process being described by a score that correlates with the automation urge. The number of scores per property is first multiplied by the corresponding factor and then summed up. The more processes are included in the checklist, the higher the probability that listed processes achieve the same score. Especially then, the weighting of the properties is recommended in order to obtain point totals that are as individual and thus as meaningful as possible.

However, by analysing your laboratory processes using the checklist, you should not exclude any laboratory processes for future automation for the time being. Depending on other factors — financial resources or technical feasibility — a process that is not very relevant at first, may be faster and easier to automate than another process that is theoretically more suitable [23]. By automating less relevant processes, resources can still be saved and invested in the non-automated process [24]. If you have applied the checklist — or as a silent reader, please continue reading Preparation of technical feasibility. If you had doubts about the validity of your automation reasons and still feel uncertain after reading this chapter, keep on with your reading to explore the numerous potential applications of automation in Possibilities*.* However, if you are sure that you currently reject laboratory automation, read as a silent reader in Preparation of technical feasibility.

## Preparation of technical feasibility 

With the help of checklist 1 (Table 1) or 2 (Table 2), processes can be identified that are reasonable to automate in a laboratory. In the next step, it will be examined whether these theoretically automatable processes are also suitable in practice, i.e. technically well suited. For this purpose, the identified processes need to be analysed and examined in more detail [19, 25]. If you have listed an entire method in checklist 1 or 2, which process step made you include the method in the checklist? Is it the numerous washing steps in ELISA or the sample preparation in chromatography? Each method can be broken down into individual steps, which of these steps can be automated depends on the financial resources and technical feasibility [26]. In order to carry out the technical feasibility check, the processes listed in checklist 1 or 2 must be prepared for this purpose. Therefore, it is essential to introduce the concept of laboratory unit operation (LUO). Please continue reading Laboratory unit operation.

### Process preparation

Automated processes are increasingly demanded by authorities and, in particular, by customers. If you see yourself in this situation, there is no space for discussion if you should automate the process but how. In this case, we need to prepare the automation step by analysing the required process [25, 27]. To prepare for technical feasibility, split your process into individual laboratory unit operations (LUO). Please continue reading Laboratory unit operation*.*

### Laboratory unit operation

By definition, a LUO is the most basic task that can be performed in the laboratory and can build a whole method by joining further LUOs [15, 28]. Admittedly, this definition is not very precise and can include different levels of detail, such as LUOs for sample processing, sample transport or data handling. If you think about what a most basic task in the laboratory is, you might think of the weighing process, pipetting or measuring samples — and of course, you are right. However, if you ask a person from the field of laboratory automation, the answer to this question will be quite different. For the specific question about the LUOs of an ELISA method, the short answer would be a sequence of the activities pipetting and shaking and the measurement of the micro-titre plate at the end (see Table 3, process no. 01). The long answer, in contrast, would not focus on the ELISA directly but would first divide the pipetting process itself into individual LUOs. This means that the pipetting process itself cannot only be considered as a single LUO, but can also be subdivided into further LUOs: (1) preparing the target vessel, (2) preparing the source vessel, (3) gripping the pipette, (4) setting the desired volume, (5) opening the tip box, (6) putting the tip on the pipette, (7) closing the tip box, (8) opening the source vessel, (9) pressing the button until the first stroke (pipette), (10) immersion in the source liquid with the pipette seat, (11) aspiration (release of the pipette button), (12) pipette tip out of the liquid, (13) pipette tip into the target vessel, (14) dispensing (up to the first stroke), (15) pipette tip out of the liquid and placed against the vessel wall, and (16) press overstroke (blow out).

**Table 3:** Identification of the LUO of the processes to be automated. To check the technical feasibility, the process to be automated must be analysed. The individual LUOs of the process should be entered in the table in the correct sequence of the process. If a checklist was used, the process numbers can be linked

Process No.	LUO 1	LUO 2	LUO 3	LUO 4	LUO 5	LUO 6	LUO 7	LUO 8	LUO 9	LUO 10	LUO 11	LUO 12	LUO 13	LUO 14	LUO 15
*01*	*Pipetting into MTP*	*Shaking*	*Washing step*	*Pipetting into MTP*	*Shaking*	*Washing step*	*Pipetting into MTP*	*Shaking*	*Washing step*	*Pipetting into MTP*	*Shaking*	*Washing step*	*Pipetting into MTP*	*Pipetting into MTP*	*Photometric measurement*
*02*	*Weighing*														
*03*	*…*	…	…	…	…	…	…	…	…	…	…	…	…	…	…

Examples are shown in italics in the table

We do not want to scare you with this example. For you, the less detailed LUO formation will be relevant for the moment. The detailed analysis, as shown in the second part of our example will be necessary when it comes to tailor-made laboratory automation or if you want to program an automatic pipetting machine, for example. However, why do we need LUOs? If you consider your process(es) to be automated, it will probably describe a method rather than a basic laboratory task (e.g. ELISA, PCR, HPLC, protein purification). Nevertheless, it is usually easier to automate individual LUOs (e.g. pipetting). Therefore, you should split your processes into the individual LUOs [29]. We have prepared Table 3 for this purpose, with an example in italics.

Table 3 can also help you to research laboratory automation systems in a later chapter. The table is intended to remind you that you can automate several LUOs with one system, automate individual LUOs and thus switch several systems in series or automate only very specific (e.g. process-critical) LUOs. The investigation of the processes will help you not to lose the focus during the research and finally to classify which of the LUOs are going to be automated by a certain system and which are going to remain manual — so you can directly identify and evaluate interfaces between manual and automated activities.

For those who used a checklist in the previous chapter, Table 3 can help validate the checklist results. You can link the process number of your checklist with the process numbers in Table 3. Do certain LUOs occur with notable frequency? Are there any LUOs that cross-process boundaries? Mark the LUOs that are good automation candidates for your lab based on their frequency and severity (from the checklist).

If you already read Possibilities, continue with Technical feasibility; otherwise, read on.

## Possibilities

Due to the different reader groups reading this chapter, it represents a kind of node in our guide. Some readers will be even more inspired to automate the processes they analysed. Rather, suspicious reader groups will be confirmed in their opinion and can continue as silent readers at the end of the chapter or change their point of view and return to Useful automated processes as “Rethinker” (see Fig. 1) to consider automation after all.

As already mentioned, it often seems that scientists have no degree of automation in their laboratory, although this is not the case according to the literature [30]. For example, Frohm et al. (2008) divide the degree of automation into 7 levels. The authors describe level 1 and 2 as the lowest level of automation in which only the muscle power of an operator or a simple, static tool is used (e.g. in microbiology level 1, pouring agar plates; level 2, plating out bacterial samples). Level 3 refers to the use of adjustable hand tools, such as pipettes, while electronic pipettes are to be classified as automated hand tools in level 4. Level 5 contains the usual laboratory equipment (e.g. centrifuge, chromatograph and spectrophotometer). Automated workstations such as pipetting machines from Tecan or Hamilton belong to level 6, while the high-end solutions, fully automated laboratory lines belong to level 7 [31].

Of course, Frohm’s (2008) classification reaches limits, so that it is not possible to give an example of every process for every level. For example, there are processes for which there is not yet a serial automation solution, and there are processes whose level of automation is standard and lower levels of automation are hardly conceivable. The second refers especially to the equipment of level 5 like centrifuges, shakers, thermocyclers and many other devices. Nevertheless, the division into seven different levels of automation shows that there are more possibilities than just the manual and the fully automated process.

Considering this classification, many scientists will certainly rate their own laboratory — depending on the process — in the range of level 1 to 5. However, this does not mean that they will continue to upgrade to levels 6 and 7 only. The upgrade strongly depends on the respective process. Thus, pipetting process, for example, does not have to be raised from level 3 to 6, but the possibilities of levels 4 and 5 should be considered and used to their full capacity.

The degree of automation usually grows gradually. A jump from a level 1 automation to, for example, a level 6 automation is hardly and only advisable under drastic changes of the entire company or whole laboratory [32, 33]. A progressive level of automation should always match the change in activities in the laboratory.

Imagine you are pipetting a biochemical assay with standard single- and multichannel pipettes (level 3) and you purchase an automated pipetting system (level 6) to automate this assay. Does the throughput justify the purchase of this workstation? Do you have enough material to fill the automated pipetting system [23]? Will you be able to measure the throughput of automated pipetted microplates on the analyser in a timely manner and process the resulting data [34]?

In most cases, the purchase would not be worthwhile. However, if you have a high throughput and it tends to increase more, the purchase should be considered [35]. An influential factor why laboratory automation is not established in every laboratory are the financial resources [2, 36]. Just as the advantages of laboratory automation — such as reproducibility and increased efficiency — are associated with automation systems, so are the high investment and follow-up costs [37]. However, nowadays various approaches can improve the degree of automation in the laboratory. Considering the automation levels 1 to 7, there will probably be no scientist among the readers who has the budget for a customized level 7 fully automated laboratory line. The costs of these laboratory lines are not only immense; they also require economic as well as building-related planning and are usually supervised by the automation system vendor [30]. However, it is crucial to consider the specific requirements of your process in terms of its level of automation. If your process is very critical in terms of factors such as timing and adherence to incubation times, it is even more important to avoid a lower level of automation.

Read on Technical feasibility if you have already decided on automation and have identified and analysed your processes (Goals to Preparation of technical feasibility). If you were unsure or initially rejected laboratory automation and changed your mind after this chapter, please go back to Useful automated processes to find your preferred automation process and check your motivation for automation (group of “rethinker”). If you think you should not automate and reject laboratory automation, you can continue reading this article as silent reader in Technical feasibility.

## Technical feasibility

Nowadays, almost every process can be automated (considering do-it-yourself or tailor-made solutions) [38]. The question that arises is with what effort (costs, person-hours, construction or production) is the automation solution associated [10]? This chapter is about to check whether automating your process by commercial systems is technically feasible.

Obviously, it is impossible to give you tailored advice in this feature article. The possible processes in a laboratory are far too specific and wide-ranging, so you will only find an approach in decision tree 1. The decision tree will ask you to do some literature research for commercial systems as a first step. This step may take some time. You can start your literature research on the Internet and look through the portfolio of known vendors or get in contact with other scientists and exchange experiences. Certainly, visits to exhibitions are helpful to get an insight into the possibilities of laboratory automation. Based on your research, you will be guided along the decision tree. Silent readers can continue reading Process requirements.

## Process requirements

Process requirements and its subchapters describe the detailed suitability check of the system you identified while your research (decision tree 1). If at any time you conclude that the system is not suitable for your needs, you are directed back to decision tree 1 to consider a different system or — if not available — a different approach.

As mentioned before, the first step is to check whether the system is generally capable of automating your process. This seems an unnecessary point for readers who have selected a system specialized for their LUO/process. Nevertheless, we want to remind you of the requirements of the specific process. For sensitive processes, the influence of the critical parameters should be reduced by automation. The robustness of the method is crucial, particularly for bioanalytical assays. It raises the question of whether it is critical to allow the process to run unattended, including overnight (e.g. stability of involved substances). If your system is an automation system that is designed for your application and the process requirements are met, please read Achieving goals.

However, for any special cases, or if you have already tested and eliminated several systems in this chapter, this is a necessary step. If you are unsure, whether the system is compatible with, for example, your chemicals, certain sample vessels or other components, check with the relevant manufacturer. If you removed any doubts and the system fits your process, continue reading in Achieving goals.

For special cases, for example, when you would misuse a system, you must determine the process requirements. This can be illustrated easiest with the help of an example. Suppose you want to automate the dispensing of agar plates in microbiology. This process seems straightforward: The process takes place under the laminar flow bench. On the one hand, you hold the glass bottle containing the liquid agar, and in the other hand, there is always an empty Petri dish into which you pour the liquid agar. The procedure must be performed quickly to avoid solidification of the agar. Using Table 3, you have identified the “pouring process” as the LUO that needs to be automated. Following decision tree 1, your research has identified dispensers that can be attached to the neck of the agar solution bottle. This allows you to add the same amount of agar solution to the plates at the push of a button, which saves time and reduces errors due to standardized volumes. Based on the description of the device, you cannot find any information that would argue against your application.

However, imagine the system in your lab: The warm liquid agar is successfully applied to a large number of Petri dishes. This must be followed immediately by a thorough cleaning process before the agar hardens. If the agar is not completely removed from the dispenser, future plates would become contaminated. Therefore, it is obligatory to establish a suitable cleaning process. Fortunately, many dispensers can also be autoclaved. This means while the dispenser is autoclaved, you have to return to the manual process if more plates need to be poured — or do you need more than one dispenser? Do the benefits of the dispenser justify the extensive cleaning of the system?

Here again, consult the manufacturer for advice on this. Another aspect that must be taken into account are environmental factors. Do you have enough space for the automation system? Are the required media and/or communication connections available [10, 18]? If you determine that the system does not meet the process requirements, return to decision tree 1 to do further research or to find another solution to your automation problem. If the system fits the process requirements, or as a silent reader, follow Achieving goals*.*

### Achieving goals

Since you verified that the system fits your process, the next step is to check if you can achieve your automation goals with this system. For this purpose, consult checklist 1 (Table 1) or checklist 2 (Table 2) again. What impact will automation have on the process? How would the system affect your throughput? Does the system have appropriate specifications to achieve the desired increase in quality [39]?

Please also consider Table 3 for this purpose. Mark the LUOs that are to be automated by the automation system in Table 3. By highlighting the automated processes in your laboratory, you can better assess the resulting consequences for your laboratory environment. In addition, the table can also support in the future when it comes to further automations of LUOs. It gives you a clear representation of the manual and automated processes and how they interact with each other.

Does automation really eliminate the source of error in an error-prone process? Will the negative characteristics of the monotonous process really be eliminated or will the characteristic shift to a related process? Imagine you acquire a laboratory automation device that automates a monotonous activity. However, you have to use device-specific laboratory ware, for example, which means that you have to transfer your samples into other vessels before using them [12]. Thus, although you have eliminated the original monotonous process, you have gained a step in your workflow that is at least as monotonous.

**Fig. 3:**
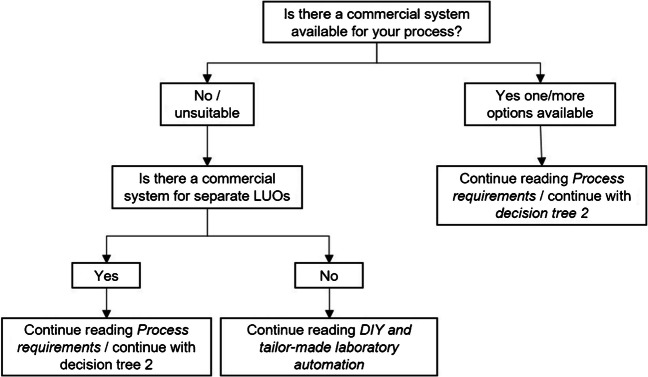
Decision tree 1: a guide for technical feasibility. The application of decision tree 1 is intended to determine whether the desired process can be automated by a commercial system. In this context, the decision tree prompts the reader to do research on a laboratory automation system

Ask yourself these and other applicable questions to critically evaluate the automation system. If you conclude the device will lead you to the automation goal, follow Economic and environmental requirements; if your device does not lead you to the desired goals, return to *decision tree 1* (Fig. 3) to resume research or pursue further possible solutions to your automation problem.

### Preparation for evaluation

The invalid reasons for laboratory automation — discussed in Theory — are not always the cause of unused automation equipment. Sometimes the reason for decommissioning the equipment is that only a few or even only one laboratory practitioner was familiar with the equipment and no one was able or willing to continue using the equipment after the trained person left the organization. As mentioned before, the decision to return unused equipment into service should be thoroughly considered, and this is regardless of whether you know the reasons for the decommissioning or not. Here, it is necessary to evaluate if the benefits of the automation device outweigh the disadvantages. To review the consequences of reviving your unused device, please continue reading in Economic and environmental requirements.

### Economic and environmental requirements

**Fig. 4:**
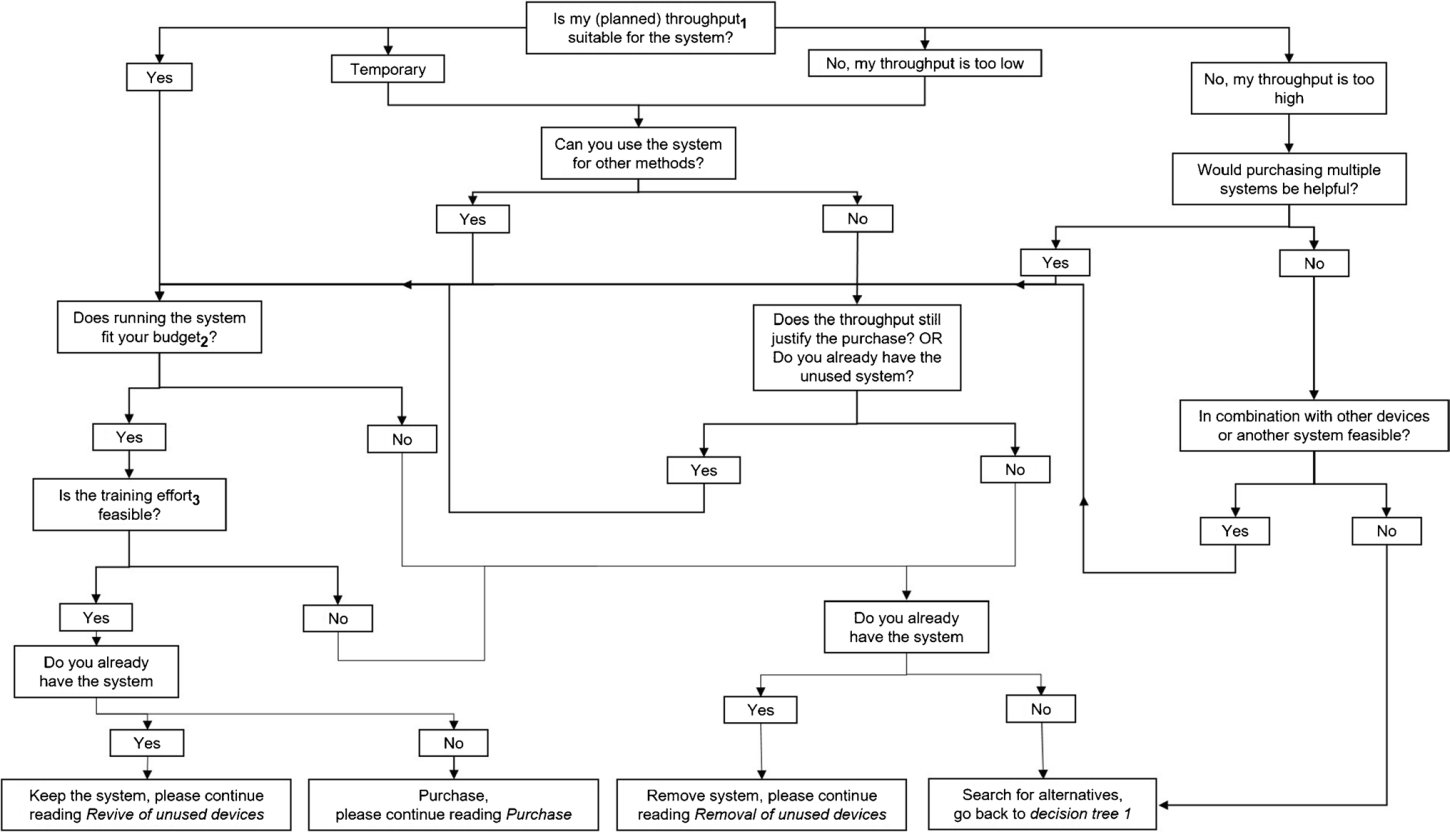
Decision tree 2: testing of the automation system for economic aspects. Depending on the applicable facts regarding throughput, budget and training effort, the decision tree helps to decide for or against the purchase of the system of choice. *1 Consideration of greater resources in terms of materials and data analysis. *2 Consideration not only of investment costs but also of follow-up costs (maintenance, new laboratory equipment, etc.). *3 Consideration of the number of employees who need to be trained

In this section, both types of systems — possible new purchases or unused equipment — will be reviewed in terms of economic aspects of your organization. For this purpose, we have developed *decision tree 2* (Fig. 4). After completing *decision tree 2*, you will conclude to purchase or revive the system, to remove an unused system or you will be guided back to decision tree 1 to review alternative systems.

In the first decision tree item, you will be asked to evaluate the system in terms of your throughput. This means the current throughput but also the throughput planned in the near future (if throughput increase is your motivation for automation) [27]. Think about the impact of an increase in throughput [22]. Do you have enough material and substances to feed the system — is this available in-house in the required quantities or is it commercially available [8]? In case it is commercially available, ask the vendor for available quantities and in which timeline those quantities can be supplied. Do you have enough personnel to make the necessary preparations and follow-ups [8]? Could there be bottlenecks somewhere in the entire process chain [37]? Ask yourself these questions and follow the decision tree accordingly.

An additional aspect related to throughput is the question of budget [27]. Can you afford the increased material requirements in the future to make the purchase worthwhile? In case of a new purchase, the budget, of course, primarily means the investment costs. In both cases — new acquisition and revival of existing equipment — follow-up costs can be generated. Does the system work with special single-use materials and what do they cost? Does the maintenance have to be done by a service technician and how often and how high are the costs? Training can also be a costly issue for complex equipment [10, 27]. Inquire how the training for the specific device might proceed. In the simplest case, the instruction manual is sufficient or — for new devices — the manufacturer provides a free introduction during delivery. However, for instruments that are more complex, a detailed knowledge of your automation device will be necessary to define or change automation processes. This can be a cost-intensive and multi-day training. Therefore, consider the training costs associated with the purchase of the equipment beforehand.

Another important aspect to consider is to look ahead and assess future prospects. While it may seem improbable at present, it is essential to contemplate the automation systems you will be acquiring in the future. This includes the concept of standardization within your team, department or even across the entire organization. On the one hand, this is important in terms of compatibility of specific labware, but also regarding the device and software logic between different vendors. Consider whether you require a certain flexibility of the devices. Many manufacturers offer hardware upgrades that come with new capabilities. Silent readers can get an overview of decision tree 2 and continue reading Decision proposals. All other readers please follow the instructions of decision tree 2.

### Decision proposals

As you have seen so far, in decision tree 2, different reader groups were ask various questions to help them determine whether a particular system is a suitable solution for their laboratory. The result can be one of four scenarios: a new device is to be purchased; an old, unused device is to be reactivated; an old, unused device is to be discarded; or — by going back to decision tree 1 — there is currently no commercial and suitable solution for the automation project. In the following chapters, you can get an idea of what to do next depending on the result of the decision tree 2 (Fig. 4). Please read Revive of unused devices.

## Revive of unused devices

In the previous chapter, you decided to recommission unused equipment, as this promises well-founded benefits for your laboratory. The first step is to become acquainted with the device. Organize the operating instructions within your laboratory or contact the manufacturer [18]. If the system has not been used for a while, first check whether it is completely functional. If not, get some advice from the manufacturer; often simple tricks can make equipment work again. Take advantage of training opportunities. Please continue reading Integration and follow-up work*.* Read Purchase as a silent reader to get an idea of what to think about when purchasing a new device.

## Purchase

Based on detailed evaluation in the last chapters, you decided to purchase at least one system to automate a specific process or certain LUOs. This process, from procurement to commissioning, can vary in complexity, as you certainly know from other laboratory equipment [40]. Usually, when purchasing a small device, the commissioning can be done by the user. Take the operating instructions to familiarize yourself with the device. For more complex devices, commissioning by a service technician or sales representative is usually part of the service [41]. In this case, please ask for a briefing. If necessary, take up further training offers and test the handling of the device. Remember, that most manufacturers offer free testing periods for their automation devices to give you an opportunity to double check on the given specifications of the device. Please continue reading Integration and follow-up work for further implementation steps.

### Integration and follow-up work

As described in previous chapters, a training plan should be established when new systems are acquired with an appropriate number of employees to train on the system [42, 43]. A rule of thumb could be that the simpler the device, the more employees should be able to use it. For more complex devices, such as pipetting machines — that are associated with a costly training programme — it may not be possible to train a larger number of employees due to financial and time factors. Trained employees are essential for the success of the automated process [44]. Therefore, you should prepare a strategy on how to train a relevant number of employees [42]. Think about absences due to illness, leaving the organization or other absences by laboratory practitioner. The more expensive and complex the system, the more important it is to prevent unforeseen system downtime. Consider also any necessary maintenance work on your automation system [37]. Small devices often have to be sent to the manufacturer; more complex devices are maintained on site by a service technician. Create a concept of how you will react to unscheduled and scheduled failures with your laboratory processes.

When the required employees are familiar with the device and its handling, plan the integration of the device. Is there important (technical) information to be considered? Use the possibility of visual notes and instruction videos to minimize any fear that colleagues may have regarding the use of automation systems [43].

Consider that (depending on which LUO you are automating) methods may not be transferred exactly as manually to the system. Here, it is important to think outside the box [45]. For example, is another pipetting scheme more practical to use the automation system? Will less buffer need to be applied in the future because the system works well with less volume, or will the volume need to be increased due to increased throughput? If necessary, consider cleaning processes or sterilization processes of the system in your laboratory routine. You will certainly need to run some test trials to validate the automated process [10].

Make sure which functions your devices cover; this could be relevant for future automations [17]. Scientists often underestimate the functions of their devices when only a small part of the functions are used daily. It is therefore worthwhile to take a look at the existing devices from time to time in order to avoid unnecessary purchases. Please continue reading Summary; silent readers may continue with Removal of unusable devices.

## Removal of unusable devices

Based on decision tree 2 (Fig. 4), you conclude that your unused automation system does not fit the needs of your laboratory and should be removed. This is an important determination. As mentioned before, laboratory space is valuable and should not be occupied by unusable equipment [46]. Therefore, you should take the effort to remove the device in your laboratory. Think about who might be interested in this equipment. Perhaps, it will serve its purpose in another laboratory within or outside your organization. Due to the high acquisition costs for such systems, there is a growing market for second-hand equipment. If you gain budget from selling the instrument, you might consider another system that could really support your daily laboratory routine. Please continue reading Summary; silent readers may continue.

## DIY and tailor-made laboratory automation

In the last chapters, you determined that there is no commercial system available to automate your process, or that the existing system(s) is/are not suitable for your needs. As previously mentioned, there is probably nothing that cannot be automated.

Perhaps the automation of existing manual devices is an interesting alternative for you. This form of laboratory automation is also available on the market and belongs to customized automation [5, 15]. The challenge here might be the effort or budget required. If a commercial device does not fully meet your requirements, you could reach out to the manufacturer. Sometimes, there are configuration options available, or the manufacturer can provide insight into whether the desired feature is currently in the planning or development stage.

In the field of do-it-yourself (DIY) automation are some publications from scientists who share their experiences and results of their own automation systems [47–51]. For scientists who are new to the field of laboratory automation, it is certainly not advisable to try to implement DIY systems in this early stage. However, depending on the complexity, it is possible that a DIY system may be suitable for you. Maybe your process can be automated with very simple means (e.g. small training robots and 3D printing) [52]. On the other hand, there are many companies that specialize in special automation solutions. These companies can advise you, propose your options and make an offer in terms of financial resources.

If the automation system is generally suitable for you, but the investment costs speak against the purchase, a look at the second hand market might be worthwhile. Due to the high investment costs, automation systems are often offered on the market again after use if, for example, projects have expired, processes change or new devices are purchased instead. Spare parts or other configuration options are often still available in the product portfolio of the manufacturers for a long time. Please continue reading Summary.

## Summary

Bioanalytical laboratories - especially medium-sized ones - are under enormous pressure to automate their processes in order to stay competitive [1, 4, 11]. This pressure is transferred to the supervisors and employees in the laboratory. Scientific practitioners are increasingly required to be familiar not only with the scientific aspects, but also with the technical aspects [7]. In this initial situation, the purchase of automation equipment without consideration leads to failure due to a lack of process analysis and planning, as well as excessive demands on the employees. The positive effects of laboratory automation in bioanalytics are undisputed - laboratories can benefit from quality improvement, time savings, increased throughput and reduced employee workload [1, 7, 12]. However, only if successfully established. The negative aspects of poorly planned automation are the waste of money and laboratory space and, most importantly, the lasting negative experience in the minds of an organization’s people [10].

With this feature article, we wanted to provide a guide that can help scientists with a laboratory background and little to no automation experience to deal with the topic in general. In case you have applied the guide (as whole or just parts), it hopefully resulted in a justified decision for or against laboratory automation in your specific case. Thus, you have a foundation for discussion if you want to initiate a purchase in your organization. At the same time, we strongly encourage you to reject laboratory automation if it is unsuitable for a process in your laboratory. We hope that we have been able to address a broad group of readers with this article and, above all, that we have been able to inspire them with the special structure of the article.
